# Price effects of calling out market power: A study of the COVID‐19 oil price shock

**DOI:** 10.1111/jems.12485

**Published:** 2022-05-19

**Authors:** Aaron Barkley, David P. Byrne, Xiaosong Wu

**Affiliations:** ^1^ Department of Economics The University of Melbourne 3010 Melbourne Victoria Australia

## Abstract

Governments often make public announcements that call into question firms' misuse of market power. Yet little is known about how firms respond to them. We study gasoline retailers' price responses to antitrust announcements shaming them for price gouging during the COVID‐19 pandemic. We identify price effects using a high‐frequency event‐study leveraging unique real‐time station‐level price data and well‐identified, discrete antitrust announcements. We find evidence of announcement effects that depend on firms' preannouncement margins and hence exposure to being publicly shamed. Public statements by antitrust questioning firms' misuse of market power can indeed contain signals that affect equilibrium outcomes.

## 1 INTRODUCTION

At the onset of the pandemic in March 2020, crude oil prices fell drastically due to a global collapse in travel and oil demand. With economies set to enter recessions, declining oil prices would provide (some) economic relief to households and businesses through lower petroleum costs. Governments quickly identified this but were concerned that oil retailers with market power, such as gasoline stations, would be slow to pass through cost savings to increase profits. Acting on these concerns, governments made public announcements pressuring oil retailers to reduce their prices to deliver cost savings to households and businesses.

History abounds with examples of governments publicly scrutinizing firms with price‐setting power dating back to Standard Oil, American Tobacco, AT&T, and Microsoft. Recently, antitrust announcements directed toward Big Pharma, Big Tech, and Big Oil have attracted significant public attention. Such history begs the question: Do firms respond to public statements made by antitrust agencies? Firms may ignore them if they view announcements as political grandstanding that does not bind the government to act. However, they may credibly signal antitrust scrutiny or rally consumers against targeted firms. Indeed, public announcements that question firms' misuse of market power may be a powerful policy option for influencing firms' behavior with the rise of social media and its ability to coordinate masses to rail against unfair business practices.

Despite the historical prevalence of antitrust announcements, there is virtually no evidence on whether they have market impacts. Identifying announcement effects has proven challenging for at least two reasons. First, finding well‐defined announcements along with (often proprietary) firm‐level data is rare. Second, announcements potentially affect all firms in an industry, making it hard to identify their causal impact on firm behavior.

In this paper, we exploit the COVID‐19 world oil price shock to study the impact of government antitrust announcements on firm behavior. Our research context, the Australian retail gasoline industry, has two features that permit such a study. First, the national antitrust authority makes discrete announcements regarding price gouging, threatening to “call out” retailers who fail to pass through falling wholesale costs at the onset of the pandemic. In Section 2 we describe these announcements and show how they propagate via the media and influence public sentiment over price gouging.

Rich data are available to study the price effects of these announcements. We discuss this second key feature of our setting in Section 3. Our data set consists of the universe of real‐time station‐level prices between January 2017 and July 2020 for a diverse cross‐section of markets ranging from metropolises like Sydney to rural monopolies in the Australian Outback. Conditional on stations' locations, they sell a homogeneous product and compete on price within their local market. This feature of the industry allows us to sharpen our focus on the price effects of antitrust announcements. Our cross‐section of markets enables us to study how these price effects vary with local market structure.

Through our combination of well‐identified announcements and real‐time price data, we can identify announcement effects using a high‐frequency event‐study. We describe this study design and present our results in Section 4. In particular, we estimate station‐level daily error‐correction models that allow for dynamic relationships between current prices with lagged prices and costs with asymmetries in station‐level price responses to negative and positive cost shocks. We also allow for retailer‐specific heterogeneity in our model's parameters to account for differences in pricing behavior across firms. We identify announcement effects as average station‐level innovations from a model's predicted price changes on announcement dates given current and lagged wholesale costs, lagged prices, and retailer types.

We obtain precisely estimated announcement effects that imply an immediate acceleration of cost passthrough that reduces margins, just as the government publicly demands. In the aggregate, announcement effects are modest. However, relatively high‐priced stations within a local market just before an announcement exhibit economically large magnitude announcement effects. Such price adjustments help these stations avoid the public scrutiny explicitly threatened by the national antitrust agency. This finding indicates that the nonbinding antitrust announcements contain payoff‐relevant information that elicits a market response.

Section 5 concludes by summarizing our results and highlighting areas for future research, which includes exploring channels for antitrust announcement effects and predicting where announcements are likely to have market impacts.

*Related literature*. Emerging research in industrial organization studies the impact of public announcements in concentrated industries. Economic theory has focused on cheap talk among firms in sustaining collusion in the presence of private information (Awaya & Krishna, 2016, 2019; Green & Porter, 1984). Few empirical studies estimate firm announcement effects on market outcomes, reflecting demanding data requirements to do so. Borenstein (2004) and Miller (2010) study price signaling and coordination among US airlines on an online price clearinghouse in the Airline Tariff Publishing Case. Aryal et al. (2021) document how US airlines signal intentions on earnings calls to coordinate on capacity levels.

By identifying market impacts of public announcements by an antitrust agency, we establish the government as a potential player in environments where firms with price‐setting power engage in signaling. We show firms immediately reduce margins in response to nonbinding antitrust announcements in a setting where price coordination is prevalent (Byrne & de Roos, 2019).^1^ Our results raise various avenues for future research on the conduct of antitrust agencies in their repeated interactions with firms, which we discuss in Section 5.

More broadly, our paper connects to empirical research on government‐led information provision and its disciplinary effects on firm behavior. Here, we most closely relate to Johnson (2020) who identifies impacts on firms' workplace safety compliance from government press releases that publicize violations of other firms. Other papers have similarly examined public disclosure effects on firms' environmental impacts (Chatterji & Toffel, 2010), drinking water quality (Bennear & Olmstead, 2008), and hygiene (Jin & Leslie, 2003). We extend this area of research by studying disciplining impacts of a government scrutinizing firms' exercise of market power publicly, specifically in failing to pass through (drastically) falling costs. Methodologically, the high‐frequency event design we use to identify announcement effects connects our paper to prior research on identifying the impact of monetary policy announcements on asset markets (Nakamura & Steinsson, 2018).

Finally, we contribute to empirical research on the retail gasoline industry. Within this literature, we most closely connect to previous studies on price coordination and tacit collusion (Byrne & de Roos, 2019; Lemus & Luco, 2021; Lewis, 2012; Wang, 2009), and cost passthrough and market structure (Bachmeier & Griffin, 2003; Borenstein & Shepard, 2002; Byrne, 2019; Deltas, 2008; Lewis & Noel, 2011; Verlinda, 2008).

## 2 COVID‐19 AND PUBLIC CONCERNS OF PRICE GOUGING

### 2.1 Context

Our setting is the retail gasoline industry in Australia. As in many countries, asymmetric retailers characterize the industry.^2^ Four brands—BP, Caltex, Coles, and Woolworths—dominate the industry. The latter two retailers also dominate supermarket retailing nationally. In the state of New South Wales (NSW), the four retailers operate 12%, 15%, 8%, and 8% of gasoline stations. The national antitrust authority, the Australian Competition and Consumer Commission (ACCC), monitors the industry and has historically pursued litigation against retailers for tacit price coordination and price‐fixing.

World oil prices are the main component of cost that affects retail prices day‐to‐day. The Singapore MOGAS 95 (Unleaded) oil price index is the crude oil benchmark for overseas fuel tankers delivering crude oil to the country's wholesale supply terminal gates. After on‐shore refining at a gate, the wholesale “terminal gate price” (TGP) for gasoline is determined. The TGP is a spot price at a given supply terminal based upon the current crude oil price and includes taxes, fixed fees such as insurance, shipping and wharfage fees, and markup for wholesalers. The TGP is the price retailers pay for the fuel that they sell to end‐consumers. Wholesale cost fluctuates day‐to‐day with the Singapore MOGAS 95 index. Retailers and wholesalers negotiate other wholesale cost factors at much lower (e.g., yearly) time frequencies.

**Figure 1:**
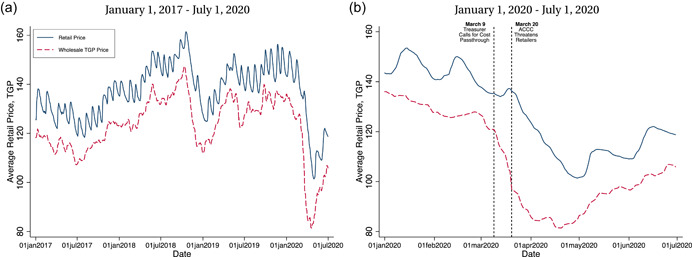
Retail and wholesale prices. [Color figure can be viewed at wileyonlinelibrary.com]

Central to our analysis is the shock to world oil prices due to COVID‐19. The global collapse in ground and air transportation associated with the pandemic sees oil prices collapse between March and May 2020. Figure 1 highlights this. Panel (a) of the figure plots the TGP and average retail price for NSW during January 1, 2017–July 1, 2020. Retail price levels track with the TGP over time. The panel also highlights cyclicality on average retail prices over time. Sydney‐based stations drive this cyclicality, which we discuss further below.

Panel (b) of Figure 1 zooms in on January 1, 2020–July 1, 2020, during the oil demand shock. During this period, the TGP falls by 40%, from 136 cents per liter (cpl) on January 1, 2020, to 81 cpl on April 20, 2020. Retail prices fall similarly.

### 2.2 Government announcements arising from COVID‐19

We study the impacts of two government antitrust announcements in the industry in March 2020. Panel (b) of Figure 1 highlights the timing of these events with vertical dashed lines.

#### 2.2.1 Monday, March 9: Treasurer calls for cost passthrough

On the afternoon of Friday, March 6, Saudi Arabia and Russia disagree on oil production cuts to stabilize world oil prices.^3^ In response to Russia's unwillingness to cut supply, Saudi Arabia cuts its prices by 10% the next day, further contributing to a subsequent oil price collapse.

These events occur overnight on Saturday in Australia (i.e., in the early morning). First thing on the morning of Monday, March 9, the Federal Government immediately implores retailers to pass through any subsequent wholesale cost reductions. The government highlights the ACCC's role in monitoring retailers' conduct and publicly shaming retailers who fail to pass through wholesale cost reductions. In particular, the Federal Treasurer and opposition party Shadow Treasurer both make public statements on Monday, March 9 that garner national news:^4^

I spoke to Rod Sims the head of the Australian Competition and Consumer Commission to re‐emphasise holding the oil retailers to account and ensure that Australians get the benefit of lower oil prices. […] The ACCC plays a monitoring role with respect to prices at the bowser and they have ensured me that they will retain their vigilance, but that they will also be calling out any energy companies that don't pass on the reduction in the wholesale price to the consumer. —Treasurer Hon. Josh Frydenberg MP, March 9, 2020

The petrol retailers in this country should not be taking us for mugs by hanging on to these substantial reductions in the fuel price. […] This is not a political issue. Every member of Parliament calls on the petrol retailers to do the right thing, to pass on these price reductions. —Shadow Treasurer Hon. Jim Chalmers MP, March 9, 2020

#### 2.2.2 Friday, March 20: ACCC publicly shames retailers

Despite the calls for cost passthrough, it simply did not happen in the eyes of the government. Ten days later, the ACCC Chair publicly shames gasoline retailers for not passing through wholesale cost savings. There is also again a threat to publicly call out individual retailers who fail to reduce their prices. The announcement again makes national news:^5^

Their prices are ridiculous. Consumers know the world price [of oil] has come down and the Australian economy needs these prices to come down, so bring them down. If they don't, we'll start calling them out. —ACCC Chair Rod Sims, March 20, 2020

### 2.3 How could the announcements influence retail pricing?

#### 2.3.1 Immediately raising awareness over high prices

Announcement effects may arise through a *static channel* if consumer awareness over gasoline pricing changes instantaneously with the ACCC's announcements and related news media coverage. In this case, consumers may perceive gasoline prices as being too high relative to their expectations, which can induce a search response (Lewis & Marvel, 2011), or cause them to punish stations they perceive as pricing excessively high (Rotemberg, 2011).^6^ Notably, the static channel implies an immediate change in payoff‐relevant information for firms. In what follows, we thus interpret announcements as nonbinding signals, rather than cheap talk, due to the possibility of immediate effects on consumer demand.

#### 2.3.2 Threatening to shame retailers

The March 9 and 20 announcements reveal the government's intention to publicly shame retailers who fail to pass through falling wholesale costs. Why might retailers be concerned about these (nonbinding) announcements? Publicly advertising price differences across retailers could have demand‐side impacts through changes in consumer search strategies or reputational effects that harm retailers' brand value. Such changes in demand could impact relatively higher‐priced firms' market shares and profits if those firms do not adjust their prices and the government carries through on its threats.^7^ In this way, the threat of being singled out in the future represents a *dynamic channel* through which announcements have an impact today.

In the context of the COVID‐19 oil price shock, being singled out for not passing through lower wholesale costs could also reveal a lack of fairness during one of the worst economic crises in history. Indeed, the March 9 announcements appeal to fairness (“do[ing] the right thing”) in reducing retail prices to ensure retailers share gains from falling world oil prices with consumers. Theories of fairness and pricing (Rotemberg, 2011) predict that negative consumer sentiment that comes with perceived unfair pricing practices can also lead retailers to cut their prices in response to a threat of being publicly shamed in the future.

### 2.4 Public interest in collusion and price gouging

**Figure 2:**
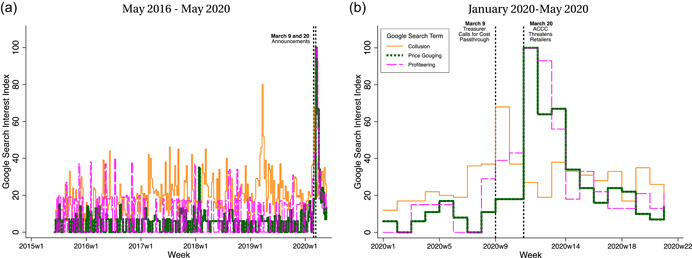
Google search interest over time keywords: “Collusion,” “Price Gouging,” and “Profiteering.” ACCC, Australian Competition and Consumer Commission. [Color figure can be viewed at wileyonlinelibrary.com]

While we are unable to assess their quantitative relevance of the static and dynamic channels,^8^ data from Google Trends provide an indication of consumer sentiment around the announcements. Figure 2 plots the Google Trends keyword search interest measure from May 2016 to May 2020 for “collusion,” “price gouging,” and “profiteering” (popular local vernacular) in Australia. Panel (a) plots the data for the entire period, showing interest in these terms reaches unprecedented levels in the first half of 2020. We zoom in on the January to June 2020 period in panel (b), which reveals “collusion” jumps the week of March 9 and “price gouging” and “profiteering” spike the week of March 20. In sum, these patterns illustrate a substantial increase in public interest in anticompetitive behavior in the weeks when the government publicly denounces retailers' lack of passthrough of falling wholesale oil prices and threatens to “call them out.”^9^ The question we now turn to is whether retailers respond to these announcements.

## 3 RETAIL PRICING

### 3.1 Data

We use a rich data set on retail gasoline prices to study the impact of the government announcements on retail pricing. In particular, we use real‐time station‐level price data for every station in NSW from January 2017 to July 2020. A platform called FuelCheck, which the state government launched on August 1, 2016, generates the data. Under state law, every time a station changes its price, it must update its price on the platform or face penalties. From our conversations with government officials, we understand that compliance is nearly perfect. The data also provide information on a station's brand and address. Throughout, we focus on ULP 91 unleaded gasoline prices, which is by far the most popular type of fuel sold.

Our sample covers January 1, 2017–June 30, 2020. We allow for a 5‐month “burn‐in” period between August 1 and December 31, 2016, to abstract from transitions in pricing immediately after FuelCheck is launched. We focus our analysis on the state capital of Sydney and 183 rural markets across the state with populations of less than 50,000 people. We use the Australian Bureau of Statistics's (ABS) geographic definition for the greater Sydney metropolitan area. Likewise, we use local market definitions from the ABS based on the location of locally isolated population clusters across the state.^10^ In total, we have 1,600,004 price observations spanning 1277 days across 635 stations in Sydney and 668 stations across the rural markets.

We match the daily wholesale TGP from Sydney to these price data. These data are available from the Australian Institute for Petrol. As discussed above, daily fluctuations in stations' wholesale costs depend on daily changes in the TGP, which varies with the Singapore MOGAS 95 crude oil price index. This aspect of our data is critical: It implies we can leverage a high‐frequency event‐study design to identify price effects of announcement effects while controlling for daily cost fluctuations using our TGP data. We return to these identification issues below.

Table 1 summarizes our data set. The left panel contains statistics for Sydney. On average, retail prices are 134.3 cpl compared with a TGP of 122.9, implying an 11.3 cpl (9%) average margin. There are 4.5 stations on average within a 1.6‐km (1‐mile) radius of a given station, including the station itself. Within the local postal areas in which firms compete, the population is 22,300 people on average, though some areas of the city exhibit much higher populations.

**Table 1:** Summary statistics.

	Sydney				Rural markets			
	Mean	Std. Dev.	Min	Max	Mean	Std. Dev.	Min	Max
*Prices (cpl)*								
Station retail price	134.3	16.6	69.9	199.9	139.6	12.1	79.8	177.9
Terminal gate price	122.9	12.6	81.4	147.3	122.9	12.6	81.4	147.3
Station retail margin	11.3	11.8	−18.2	94.9	16.6	8.4	−18.5	85.7
*Market structure*								
Total stations in a local market	4.5	2.4	1.0	12.0	3.7	3.1	1.0	20.0
HHI in terms of station shares	3888	2449	1200	10,000	4693	2845	1094	10,000
Distance from supply terminal	26.7	14.4	1.3	81.9	355.2	173.7	56.3	939.3
Population (1000s)	22.3	14.7	1.8	105.5	5.7	8.0	1.0	48.3
*Panel dimensions*								
Stations	635				668			
Dates	1277				1277			
Local markets	635				183			
Observations	774,302				825,702			

*Notes*: Sample period is January 1, 2017–July 1, 2020. In rural NSW, a local market is defined by the urban centers in the Australian Bureau of Statistics's UCLs. For Sydney, we report the number and the HHI of all stations within a 1.6‐km (approximately 1 mile) radius of a given station. Distance from supply terminal is measured in km.

Abbreviations: HHI, Herfindahl–Hirschman Index; NSW, New South Wales; UCL, Urban Centres and Locality.

The right panel shows rural markets have higher retail prices and margins on average at 139.6 and 16.7 cpl. A typical local rural market has 3.7 stations, and the sample includes 34 rural local monopolists out of 183 local rural markets. An average rural market has 5700 residents and is 355.2 km from the Sydney‐based wholesale supply terminal.

### 3.2 Urban price dynamics

**Figure 3:**
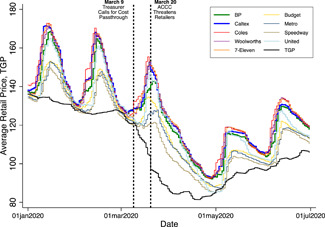
Price dynamics in Sydney. ACCC, Australian Competition and Consumer Commission; TGP, terminal gate price. [Color figure can be viewed at wileyonlinelibrary.com]

There are two distinct types of price dynamics in the data. As in many urban markets worldwide (Eckert, 2013; Noel, 2016, Chap. 16), Sydney's prices follow an asymmetric cycle whereby prices infrequently jump with runs of daily price cutting between price jumps.^11^ Figure 3 plots average daily station‐level prices, by brand, from Sydney, highlighting the cycle and differences in cycles across the brands.^12^ Stations run by the major brands—BP, Caltex, Coles, and Woolworths—along with 7‐Eleven have relatively steeper cycles compared with smaller independent retailers.^13^ Sydney's cycle has had this form since 2010, with cycle length varying around 30 days and the price jump occurring on different days of the week (Byrne & de Roos, 2019).

Relating Sydney's prices to the announcements, we make two preliminary observations from Figure 3. First, the price jumps in January and February 2020 are approximately 30 cpl (≈20% increases) and occur when retail prices approach the wholesale TGP. In early March 2020, despite an eventual drop in wholesale costs due to the OPEC‐Russia price war, the retailers continue to coordinate their regular 30 cpl price jump. The March cycle peak is when the ACCC publicly shames the retailers for not passing through falling wholesale costs. On the March 20 announcement date, average retail prices for Caltex and Coles are 155 cpl which, relative to a wholesale TGP of 95 cpl, implies a substantial 60% markup.

The second observation is that following the ACCC's announcement, we find a long cycle that breaks from the historical 30‐day cycle length. It is not until May 7 that we see another price jump occur, more than 60 days after the price jump at the start of March. Keeping with past pricing, however, the timing of this jump coincides with when retail prices approach the wholesale TGP at the beginning of May. At this point, global oil supply cuts by Russia and OPEC begin to take hold following their price war. As Figure 3 shows, wholesale prices gradually start recovering, and the price cycle trends upward with them.

### 3.3 Rural price dynamics

**Figure 4:**
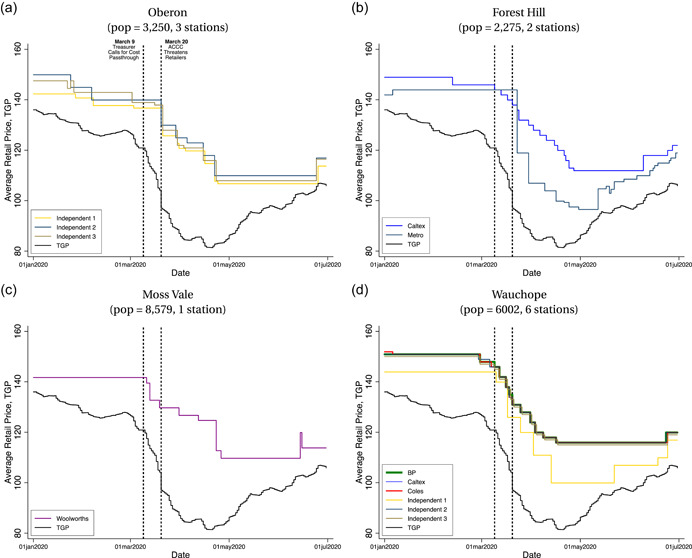
Price dynamics in rural markets. ACCC, Australian Competition and Consumer Commission; TGP, terminal gate price. [Color figure can be viewed at wileyonlinelibrary.com]

The rural markets exhibit markedly different price dynamics. To illustrate this, panels (a)–(d) of Figure 4 plot station‐level prices for four different rural markets in our sample: Oberon, Forest Hill, Moss Vale, and Wauchope. Retail prices do not cycle, and they experience multiday runs without any adjustments despite daily fluctuations in wholesale costs.

We find examples of station‐level price changes with the ACCC announcement on March 20 but not the Federal Treasurer announcement on March 9. Stations in Oberon all have immediate, nonnegligible price cuts on both dates. In contrast, in Forest Hill, only the Metro station has a substantial price cut immediately after March 20. Stations in Wauchope, a more competitive market with six stations, have frequent and small price cuts in response to falling wholesale costs during the COVID‐19 pandemic with no price adjustments immediately following either ACCC announcement.

## 4 PRICE EFFECTS OF ANTITRUST ANNOUNCEMENTS

To formally estimate stations' price responses to antitrust announcements, we use econometric models that account for dynamic relationships between stations' daily prices and costs. Using our models, we can predict what pricing behavior should look like on the day of an announcement, given current and lagged costs and lagged prices.^14^ We identify announcement effects as the departure of realized prices from our models' predicted prices on dates when the government makes public statements about sluggish cost passthrough and price gouging.

We proceed in two parts. We start with rural markets because modeling their price dynamics is straightforward. We then analyze the impact of antitrust announcements in Sydney, where it is necessary to account for asymmetric price cycles. Econometrically, this is nontrivial, but conceptually identifying announcement effects parallels our approach from rural markets.

### 4.1 Announcement effects in rural markets

We identify announcement effects in rural markets in two steps. First, we estimate the error‐correction model from Borenstein et al. (1997), a framework that has been used extensively for estimating retail gasoline cost passthrough (Eckert, 2013). The model predicts the response of station i's price on date $t,p_{it}$, to current and lagged wholesale TGP $c_{it}$ and lagged prices:

(1) $$\begin{array}{ccl} \Delta p_{it} & = & \sum_{k=0}^{49}\left(\beta_{k}^{+}\Delta c_{i,t-k}^{+}+\beta_{k}^{-}\Delta c_{it-k}^{-}\right)+\sum_{k=1}^{21}\left(\gamma_{k}^{+}\Delta p_{it-k}^{+}+\gamma_{k}^{-}\Delta p_{it-k}^{-}\right)\\ & & +\theta_{i}^{+}z_{it}^{+}+\theta_{i}^{-}z_{it}^{-}+\sum_{k=\;\text{1mar20}}^{\text{30apr20}\;}\delta_{k}1\left\{t==k\right\}+\epsilon_{it}, \end{array}$$

(2) $$z_{it}=p_{it-1}-\left(\phi c_{it-1}+\sum_{i=1}^{N}\left(\nu_{i}STATION_{i}\right)\right),$$

where $\Delta p_{it}=p_{it}-p_{it-1},\Delta c_{it}=c_{it}-c_{it-1}$, and $z_{it}$ is the residual of an ordinary least squares regression of $p_{it-1}$ on $c_{it-1}$ and a vector of station fixed effects $STATION_{i}$ for i=1,…,N.^15^ Importantly, the model allows for asymmetric short‐run price responses to positive and negative cost shocks with $\Delta c_{i,t-k}^{+}=\max\left\{0,\Delta c_{it}\right\}$ and $\Delta c_{i,t-k}^{-}=\min\left\{0,\Delta c_{it}\right\}$ with $\Delta p_{i,t-k}^{+}$ and $\Delta p_{i,t-k}^{-}$ similarly defined. The error‐correction terms, $z_{it}^{+}=\max\left\{0,z_{it}\right\}$ and $z_{it}^{-}=\min\left\{0,z_{it}\right\}$, allow for asymmetric long‐run transitions in prices, depending on whether a station's price is currently above or below its long‐run level.^16^ We allow for station‐level heterogeneity in these transitions through the $\theta_{i}$ coefficients in Equation (1). We compute cluster bootstrap standard errors (Cameron et al., 2008) at the local market level from a distribution of B=200 bootstrap coefficient estimates for the parameters in (1) and (2). Doing so allows for arbitrary correlation in the model's unobservables across stations within a local market at a point in time, and for a local market over time.

#### 4.1.1 Identification

The parameters of interest in (1) are the $\delta_{k}$'s. These coefficients are on a set of calendar date dummies for March 1–April 30, 2020. They quantify average station‐level departures in predicted price adjustments on each date within this period. We are particularly interested in the $\delta_{k}$ estimates around the dates of the March 9 and 20 announcements. $\delta_{k}$ estimates around these dates identify any passthrough‐accelerating effects of an announcement assuming that: (1) our error‐correction model accounts for the impact of current and lagged daily wholesale costs and lagged retail prices on current price changes; (2) announcements are unanticipated; and (3) there are no other date‐specific contemporaneous shocks that affect retail pricing.

The completeness of our price data, and the fact that the wholesale TGP is the main cost affecting stations' daily pricing decisions, gives us confidence that our model can account for station‐level adjustments to daily wholesale costs. Further, as Figure 1 shows, our long, 3‐year panel of retail and wholesale prices includes substantial increases and decreases in wholesale costs. Such variation provides necessary identifying variation for our model that allows us to predict price responses for a wide range of daily wholesale cost shocks in predicting counterfactual prices in the absence of announcements on the announcement dates.^17^

*COVID*. During the pandemic there is an aggregate shock to gasoline demand due to households being locked down in their homes. What is important for the identification of announcement effects is that, as per assumption (3), there are no high‐frequency (e.g., daily) lockdown‐related shocks on announcement dates that might affect demand. Supporting Information Appendix A provides details on the evolution of COVID‐related government policy during the sample period, confirming that on March 9 and 20 there are no COVID‐related announcements of lockdowns or imposition of lockdowns by the NSW state government. The Supporting Information also shows that while traffic volumes are much lower relative to pre‐COVID levels during the sample period, they are stable on the announcement dates that we are interested in.

As a further check on potential impacts of COVID‐related confounds on identification, we also present estimation results where we explicitly include three COVID controls in Equation (1). These controls are: (1) $\Delta Cases_{t-k}$, the change in state‐level COVID‐19 case counts in NSW on date t; (2) $\Delta Restrictions_{t-k}$, a dummy variable equaling one if there is a change in COVID‐19 restrictions on date t ^18^; and (3) $\Delta lnTraffic_{it-k}$, the change in the logarithm of the traffic volume near station i.^19^ For $\Delta Cases_{t-k},\Delta Restrictions_{t-k},\Delta lnTraffic_{it-k}$ we, respectively, include k=0,…,7,k=0,…,3, and k=0,…,14 lags in our robustness checks.

### 4.2 Rural market results

Column (1) of Table 2 presents our baseline results. Respectively, the March 9 and 20 announcements yield excessive price cuts of 0.18 and 0.54 cpl relative to what our model predicts.^20^ While precisely estimated, the magnitudes of the announcement effects are relatively small: respectively, they are 1.0% and 3.2% of average daily margins of 16.7 cpl across our rural markets. The column (2) estimates in the table show that accounting for our COVID controls has little impact on these estimates, supporting our high‐frequency event‐study research design.

**Table 2:** Price effects of antitrust announcements in rural markets.

	(1)	(2)	(3)	(4)	(5)	(6)
March 9	−0.18***	−0.15**	−0.15**	−0.14*	0.37	0.41
	(0.06)	(0.07)	(0.07)	(0.08)	(0.25)	(0.52)
March 9 × Above median price			−0.08	−0.01	−0.13	−0.07
			(0.11)	(0.18)	(0.10)	(0.19)
March 9 × Number of stations					−0.02	−0.01
					(0.02)	(0.04)
March 9 × HHI					−1.13*	−0.69
					(0.62)	(0.91)
March 9 × Dist. from terminal gate					−0.12	−0.79
					(0.38)	(0.53)
March 20	−0.54***	−0.58***	−0.24**	−0.17	−0.58	−1.60
	(0.11)	(0.22)	(0.10)	(0.19)	(0.48)	(1.16)
March 20 × Above median price			−1.03***	−1.30***	−1.02***	−1.26***
			(0.26)	(0.46)	(0.26)	(0.46)
March 20 × Number of stations					−0.00	0.06
					(0.03)	(0.10)
March 20 × HHI					−0.12	1.95
					(0.70)	(1.29)
March 20 × Dist. from terminal gate					1.11*	1.52
					(0.66)	(1.13)
COVID controls						
$\Delta Cases_{t-k}$	N	Y	N	Y	N	Y
$\Delta Restrictions_{t-k}$	N	Y	N	Y	N	Y
$\Delta lnTraffic_{t-k}$	N	Y	N	Y	N	Y
Observations	795,022	352,793	795,022	352,793	795,022	352,793
*R* ^2^	0.06	0.06	0.06	0.06	0.06	0.06

*Notes*: Regression coefficients between date of sample and market structure interactions included in Equation (1). See the text for a discussion of the model and Tables B.1 and B.2 in Supporting Information for the full set of regression coefficient estimates from Equations (1) and (2). Standard errors clustered at the local market (UCL) level.

Abbreviation: HHI, Herfindahl–Hirschman Index.

*** p<0.01

** p<0.05

* p<0.1.

Does the magnitude of announcement effects vary with a station's location within its local market's price distribution just before an announcement? To investigate this, we include a set of interaction variables in (1) that interact the date dummy variables for March 9 and 20 with a dummy variable equaling one if station i's price is above the median price in its local market the day before an announcement. The inclusion of this dummy and its interaction with announcement date dummies is motivated by: (1) language used by the government in its announcements in threatening to call out high‐price retailers; and (2) the ACCC's previous use of within local market price rankings to publicly identify Coles as the highest‐priced retailer nationwide in October 2019. The announcements may signal relatively high‐priced stations within local markets that the government will call them out if they do not reduce their prices. Following announcements, consumers may also become particularly sensitive to avoiding high‐priced stations within their local market. Both channels lead us to expect high‐price stations within local markets to exhibit relatively large announcement effects.^21^

We also include interaction variables in (1) that interact the date dummy variables for March 9 and 20 with a vector of market structure variables $X_{it}$. The variables in $X_{it}$ include the number of stations in a station's local market, Herfindahl–Hirschman Index (HHI) in terms of retailers' station shares within a local market, and a station's distance from the terminal gate in Sydney.^22^

Columns (3) and (5) of Table 2 present our heterogeneous announcement effects results. Our main finding is that the larger March 20 announcement effect of −0.54 stems from stations' position in the price distribution just before the announcements. Our column (3) estimates reveal that stations whose prices are above their local market's median price the day before these announcements have excessive price cuts of 1.03 relative to stations pricing at or below their market's median price. The magnitude of the overall announcement effect for these stations on March 20 of 1.27 is large at 8% of average rural market profit margins. This finding is consistent with retailers viewing the threat of being called out in the ACCC's March 20 announcement as being credible or the announcement having an immediate impact on demand. In either case, the ACCC's public announcements had an equilibrium impact on retailers' pricing decisions. None of these conclusions change when we control for local market structure in column (5), nor when we include COVID controls in columns (4) and (6).^23^

Notably, the March 20 announcement effect is greater than the March 9 effect. It is possible that the March 9 announcement by the Federal Treasurer “primed” consumers or stations and the subsequent March 20 announcement effect by the ACCC Chair. In other words, there may be an interaction between the announcements, even if each announcement effect lasts for only a few days (as we show momentarily). It is also possible that threats from the national antitrust authority on March 20 carry more weight than those from the Treasury on March 9. While we cannot test for interaction effects between the announcements nor the impact of different parts of government making announcements, they are contextual factors to be mindful of when interpreting differences in the magnitudes of the March 9 and 20 announcement effects.

#### 4.2.1 Evolution of announcement effects

**Figure 5:**
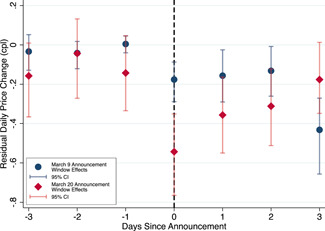
Announcement effect evolution in rural markets. [Color figure can be viewed at wileyonlinelibrary.com]

How do the announcement effects evolve? To examine this, Figure 5 presents an event time graph which plots the $\delta_{k}$ estimates and their 95% confidence intervals for a 3‐day window around the March 9 and 20 announcements. We interpret the graph with some caution as moving away from the announcement dates departs from our high‐frequency event‐study research design, potentially introducing confounding time‐varying unobservables on other dates. We believe the graph yields two notable results with this caveat in mind. First, there are no trends in the $\delta_{k}$ estimates in the 3 days before each announcement. These estimates indicate an absence of time‐varying unobserved shocks just before the March 9 and 20 announcements, which supports our research design.

The second result is that antitrust announcement effects appear short‐lived, lasting for just 2 days following each announcement. The upward trending and significantly $\delta_{k}$ estimates 2 days after the March 9 and 20 announcements reveal this result. As per our discussion of potential mechanisms, this evolution of the announcement effects potentially reflects: (i) consumers' short‐lived awareness of high gasoline price‐cost margins or heightened search; or (ii) short‐run pricing adjustments by rural stations to avoid being publicly identified by the national antitrust authority for their excessively slow cost passthrough.

#### 4.2.2 Retailer‐specific announcement effects

We are also interested to see if particular firms drive the announcement effects. To study this, we estimate retailer‐specific announcement effects (i.e., $\delta_{k}$'s) by including interactions between the announcement date and retailer dummy variables in Equation (1).

Table B.3 in the Supporting Information presents these auxiliary results. All four dominant retailers—BP, Caltex, Coles, and Woolworths—exhibit statistically and economically significant announcement effects. However, these retailers stations' positions in price distributions within local markets just before the announcements drive these effects.^24^ Independent retailers also exhibit nonnegligible announcement effects, even after controlling for their stations' locations within their local markets' price distributions. In sum, announcement effects exist for dominant and independent across the rural markets and depend on their stations' locations within local price distributions preannouncement. Announcement effects do not reflect systemic price adjustments among a subset of particular retailers in rural markets.

### 4.3 Announcement effects in Sydney

We now turn to announcement effects in Sydney. Two features distinguish this analysis from the rural markets analysis. First, we treat Sydney as a single large, interconnected market. Second, Sydney gasoline exhibits asymmetric price cycles that make the error‐correction model of Equations (1) and (2) unsuitable. Price cycles have prolonged runs of small price cuts, the “undercutting” phase, followed by an abrupt “relenting” phase characterized by sudden price jumps.

To account for the cycling nature of prices, we follow Lewis and Noel (2011) and use a Markov switching regression to model the dynamics of retail pricing and costs at the station‐level.^25^ In the model, a station is either in the undercutting (s=0) or relenting (s=1) phase of the cycle. Transitions between these states are modeled according to a phase s‐specific logit framework with latent utility:

(3) $$\begin{array}{ccl} y_{it}^{*} & = & \beta_{0}^{s}+\beta_{1}^{s}m_{it-1}+\beta_{2}^{s}c_{t}+\sum_{j=1}^{J}\alpha_{j}^{s}1\left\{m_{it-1}\leq M_{j}\right\}+\sum_{k=1}^{K}\gamma_{k}^{s}\Delta c_{t-k}\\ & & +\sum_{k=\;\text{1mar20}}^{\text{30apr20}\;}\delta_{k}1\left\{t==k\right\}+\eta_{i}^{s}+\tau_{m\left[t\right]}^{s}+\xi_{y\left[t\right]}^{s}+\varepsilon_{it}^{s}, \end{array}$$

where $m_{it}=p_{it}-c_{it}$ is station i's margin on date $t,1\left\{m_{it-1}\leq M_{j}\right\}$ is an indicator function equaling one if the margin is below threshold $M_{j}$,^26^ and $\tau_{m\left[t\right]}^{s}$ and $\xi_{y\left[t\right]}^{s}$ are month and year fixed effects. As in our rural market model, the $\delta_{k}$ coefficients on a set of date dummies between March 1 and April 30, 2020 allow us to investigate abnormalities in state transitions around the announcement dates. The error term, $\varepsilon_{it}^{s}$ is assumed to be Type‐1 Extreme Value distributed implying that the probability a station switches from phase s to the relenting (s=1) phase has the familiar logit form:

(4) $$\lambda_{it}^{s0}=\frac{\text{exp}\left(W_{it}\Psi^{s}\right)}{1+\text{exp}\left(W_{it}\Psi^{s}\right)},\;\;s=0,1,$$

where $W_{it}$ and $\Psi^{s}$ are the vectors that, respectively, contain all the right‐hand side variables and phase‐s specific coefficients in (3).

The presence of a large price jump at a station in the data represents a one‐to‐one mapping to the relenting phase. Price jumps in the data thus allow us to specify whether station i is in the relenting or undercutting phase on date t over our January 1, 2017–July 1, 2020 sample period.^27^ Following Byrne and de Roos (2019), we classify station i as transitioning to the relenting phase on date t if $\Delta p_{it}\geq 5$ cpl; all of our results are robust to the threshold used.^28^ Supporting Information Appendix B reports estimates and standard errors clustered by postcode for the parameters in Equations (3)–(5). Our standard errors account for spatial correlation in the model's errors across retailers within local areas of Sydney, as well as persistence in the postcode‐level errors over time.^29^

Conditional on being in phase s, prices evolve according to the following equation:

(5) $$\begin{array}{ccc} \Delta p_{it}^{s} & = & \beta_{1}^{s}m_{it-1}^{s}+\beta_{2}c_{it}^{s}+\sum_{k=1}^{K}\gamma_{k}^{s}\Delta c_{t-k}^{s}\\ & & +\sum_{k=\;\text{1mar20}}^{\text{30apr20}\;}\delta_{k}^{s}1\left\{t==k\right\}+\eta_{i}^{s}+\tau_{m\left[t\right]}^{s}+\xi_{y\left[t\right]}^{s}+\epsilon_{it}^{s}, \end{array}$$

where all other variables are as in Equation (3). Again, we emphasize the inclusion of date dummies between March 1 and April 30, 2020 allows us to examine announcement effects in firms' pricing during the relenting and undercutting phases of the cycle. In sum, $\delta_{k}$ coefficients with high magnitudes in Equations (3) and (5) indicate large deviations from the expected outcome after conditioning on cost, margin, time, and station‐specific variables. Hence, coefficients on announcement days that differ substantially from those just before the announcement date constitute an announcement effect.

#### 4.3.1 Identification

Our identifying assumptions for announcement effects are analogous to those from rural markets. We assume: (1) our Markov switching regression model accounts for the impact of current and lagged daily wholesale cost and lagged retail prices on current prices; (2) announcements are unanticipated; and (3) no other date‐specific shocks affect local retail pricing. Supporting Information Appendix A, which recall describes the COVID‐related timeline and provides evidence on (a lack of) shocks to government policy or daily traffic shocks on the announcement dates support assumption (3). As a check on COVID‐related confounds, we present results that include the COVID controls from our rural stations analysis above (with analogous lag structures), $\Delta Cases_{t-k},\Delta Restrictions_{t-k}$, and $\Delta lnTraffic_{it-k}$ in our Markov switching regression model for Sydney stations.

Given the nature of price leadership and coordination with price cycles (Byrne & de Roos, 2019; Lewis, 2012), our first assumption may be of concern if pricing conduct changes during the COVID period. Such a change could imply that a Markov switching regression model based on pre‐COVID data may lead to poor price predictions of “standard” pricing behavior. Accordingly, we include a post‐COVID dummy variable to account for changes in mean pricing and state transitions during the COVID period. In addition, Table B.5 in Supporting Information presents results from other specifications as robustness checks, including a version of the model estimated on the COVID period only, yielding similar results.

### 4.4 Sydney results

Table 3 presents the announcement effect estimates for Sydney from Equations (3) to (5).^30^ Panel (a) presents our baseline effects for pricing during the undercutting phase, relenting phase, and state transitions between the phases. Focusing first on the undercutting phase of the cycle, where the bulk of observations lie on each announcement date, the column (1) estimates yields nonnegligible, statistically significant announcement effects on retail pricing of 0.53 and 1.48 cpl on March 9 and 20, respectively. Column (2) shows that these effects remain after including COVID controls in our model, albeit they are smaller in magnitude at 0.12 and 1.13 cpl, respectively.

**Table 3:** Price effects of antitrust announcements in Sydney.

	Undercutting price		Relenting price		State transitions	
	(1)	(2)	(3)	(4)	(5)	(6)
*Panel (a): Main effects*						
March 9	−0.53***	−0.12**	16.01***	4.69***	−0.06	−1.20***
	(0.10)	(0.05)	(1.87)	(1.77)	(0.67)	(0.30)
March 20	−1.48***	−1.13***	11.35***	−6.03***	6.02***	0.73*
	(0.28)	(0.29)	(1.88)	(1.91)	(0.68)	(0.40)
COVID controls						
$\Delta Cases_{t-k}$	N	Y	N	Y	N	Y
$\Delta Restrictions_{t-k}$	N	Y	N	Y	N	Y
$\Delta lnTraffic_{t-k}$	N	Y	N	Y	N	Y
*R* ^2^	0.25	0.25	0.93	0.93	0.18	0.17
Observations	726,325	679,516	31,026	28,989	712,632	678,933
*Panel (b): Effects of price level and local competitors*						
March 9	−0.60***	−0.06	14.29**	0.68	−2.41**	−3.85***
	(0.12)	(0.11)	(5.81)	(7.09)	(1.01)	(0.92)
March 20	−0.83	−0.36	12.94***	−3.66	6.43***	1.35**
	(0.53)	(0.61)	(2.82)	(2.71)	(0.83)	(0.64)
Above median price	−0.38***	−0.38***	−0.42***	−0.16	−0.81***	−0.81***
	(0.02)	(0.02)	(0.15)	(0.15)	(0.06)	(0.06)
March 9 × Above median price	0.05	0.00	4.01	5.63	2.14***	2.45***
	(0.10)	(0.11)	(4.47)	(5.77)	(0.48)	(0.55)
March 20 × Above median price	−2.52***	−2.56***				
	(0.32)	(0.37)				
Number of stations within 1 mile	−0.15***	−0.04***	0.35	−0.75***	0.04***	0.08***
	(0.01)	(0.01)	(0.28)	(0.27)	(0.00)	(0.00)
March 9 × Number of stations	−0.00	−0.01	−0.12	−0.21	0.32***	0.30**
	(0.02)	(0.02)	(0.40)	(0.43)	(0.11)	(0.12)
March 20 × Number of stations	0.08	0.11	−0.30	−0.34	−0.11	−0.15
	(0.09)	(0.10)	(0.38)	(0.42)	(0.11)	(0.12)
COVID controls						
$\Delta Cases_{t-k}$	N	Y	N	Y	N	Y
$\Delta Restrictions_{t-k}$	N	Y	N	Y	N	Y
$\Delta lnTraffic_{t-k}$	N	Y	N	Y	N	Y
*R* ^2^	0.26	0.26	0.93	0.93	0.18	0.18
Observations	726,325	679,516	31,026	28,989	712,369	678,719

*Notes*: There were no stations whose previous day prices were above the median market price in the relenting phase on March 20. Standard errors clustered at the postcode level. *R* ^2^ denotes pseudo‐$R^{2}$ for state transition models.

*** p<0.01

** p<0.05

* p<0.1.

The pooled estimates in columns (1) and (2) mask differences in announcement effects across stations in different parts of the preannouncement price distribution, just as we found for rural markets. In panel (b) of Table 3, we allow announcement effects to be moderated by whether a station is above the median price across Sydney the date before the announcements, and by the number of stations 1‐mile from a given station.^31^

Columns (1) and (2) in panel (b) of Table 3 reveal a significant announcement effect on undercutting among relatively high‐priced stations on March 20. Our baseline estimates in column (1) of panel (b) show that prices fell by 2.52 cpl more than expected among high‐priced stations following this announcement. These represent substantial pricing abnormalities at 22% of the 11.3 cpl average retail margin in Sydney.^32^ These findings reinforce our main result that firms respond to antitrust announcements when exposed to potentially being called out for being higher priced compared with rivals. By contrast, local competitiveness within a mile of a given station does not moderate announcement effects. The column (2) estimates in panel (b) show that including COVID controls in the model has a minimal impact on these results.

Our station‐level results regarding pricing during the relenting phase and state transitions are mixed and noisier. This imprecision stems from only observing one relenting phase around the announcements, particularly on March 20. A notable finding from the statistically significant 6.02 state transition coefficient estimates in column (5) of panel (a) is that the propensity for stations to engage in a price jump on March 20 is abnormally high relative to typical price cycling behavior. The intuition for this follows from the fact that our Markov switching regression estimates, reported in Tables B.4 and B.5 of the Supporting Information, predict that price jumps become likely as retail prices approach the wholesale TGP. However, as can be seen in Figure 3, a price jump is coordinated on March 20 even though retail prices are well above wholesale costs. Our model identifies this abnormal behavior on March 20 through the coefficient estimates in columns (3) and (4) in both panels (a) and (b) of Table 3. In this sense, these estimates indicate that the timing of the antitrust announcement effects in Sydney coincides with a period where abnormal price jumps are occurring while world oil prices are collapsing and margins are high.

This timing of announcements potentially undermines the interpretation of the coefficients for pricing during the relenting phase on March 20 in columns (3) and (4) of Table 3. However, it does not affect the interpretation of the abnormal price cuts among rural stations nor Sydney‐based stations in the undercutting phase of the cycle on March 20 as announcement effects as long as the announcements are unanticipated.

#### 4.4.1 Evolution of announcement effects

**Figure 6:**
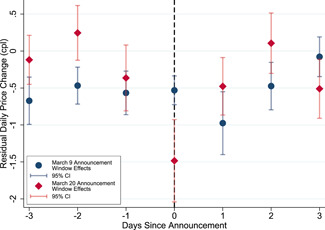
Announcement effect evolution in Sydney. [Color figure can be viewed at wileyonlinelibrary.com]

We plot the $\delta_{k}$ estimates for Sydney‐based stations for 3 days before and after March 9 and 20 to study the evolution of announcement effects. Figure 6 presents these estimates in event‐time along with their 95% confidence intervals. We again find no clear trend in the $\delta_{k}$ estimates before the announcements, validating our high‐frequency event‐study design. The post‐announcement $\delta_{k}$ estimates reveal, at most, 2 days of short‐lived announcement effects, similar to what we find in rural markets. For instance, the day after the March 9 announcement, there is a statistically significant $\delta_{k}$ that implies an excessive 0.98‐cpl price cut. For the March 20 announcement, the figure highlights an immediate announcement effect of 1.48 cpl which decays to 0.46 cpl on March 21. Short‐lived changes in consumer attentiveness to gasoline prices or retailers' conduct following the announcements potentially underlies this evolution of announcement effects.

Comparing Figures 5 and 6, we find announcement effects to be shorter‐lived for Sydney‐based stations. One explanation for this result is the difference in equilibria in rural markets and Sydney. Borrowing terminology from Noel (2008), rural markets exhibit “sticky pricing” with relatively less frequent daily price adjustments, whereas Sydney exhibits “price cycles” with constantly adjusting prices. Announcement effects among rural market stations to government announcements are potentially longer‐lived compared with Sydney stations due to these differences in day‐to‐day price rigidity under sticky pricing and price cycles.

#### 4.4.2 Retailer‐specific announcement effects

Finally, we present retailer‐specific announcement effects for Sydney in Table B.6 of the Supporting Information. We focus on stations engaged in price undercutting on announcement dates as most stations are undercutting on these dates.^33^ Overall, we find mixed results across the retailers, with March 20 being the date where the major retail chains in Sydney (7‐Eleven, BP, Caltex, Woolworths, and Coles) exhibit large and statistically significant announcement effects.

However, Table B.6 further shows that once we allow announcement effects to depend on whether a station is pricing above the median price in Sydney the day before an announcement, retailer‐specific announcement effects become statistically insignificant. As we found in rural markets, station‐level price responses in Sydney primarily depend on individual stations' position in the price distribution preannouncement and not a station's brand.

## 5 CONCLUSION

Antitrust agencies have long monitored industries, often evoking public scrutiny over firms' misuse of market power through announcements that do not bind agencies to act. We have provided a first empirical study of whether firms respond to public, nonbinding threats from an antitrust authority. In particular, we identified margin‐reducing price effects from government announcements shaming and threatening gasoline retailers for failing to pass through falling world oil prices at the onset of the COVID‐19 pandemic.

Our findings highlight the signaling role of antitrust authorities in concentrated industries. Indeed, the focus of emerging theoretical and empirical research on cheap talk and public signaling in settings where firms have market power has thus far focused on firms' ability to coordinate on pricing (e.g., Aryal et al., 2021; Awaya & Krishna, 2016, 2019). Our results show that nonbinding announcements by governments can also alter equilibrium price paths. We have discussed static and dynamic channels for such price effects. Statements by antitrust agencies can affect consumer awareness over prices and search behavior and thus firms' pricing. In addition, threats of future regulatory action can also impact firms' willingness and ability to raise prices. Data on firm and consumer responses to antitrust announcements, and models of reputation‐building by antitrust authorities, are needed to disentangle channels through which government announcements affect equilibrium outcomes in future research.

Such research can inform predictions of where government announcements are likely to have market impacts. These predictions, in turn, depend on how announcements interact with economic primitives in markets (e.g., preferences, beliefs, technology, and institutions). As we have alluded to, information frictions likely have a first‐order impact. Industries characterized by search frictions or opaque prices such as retail gasoline, healthcare, electricity, or household finance may be conducive to announcements creating procompetitive effects by stimulating and informing consumer search behavior. Technologies such as price comparison platforms may augment government announcements by enabling search and punishment of high‐priced firms by consumers.

## Supporting information

Supplementary Information

## ENDNOTES

1 Clark and Houde (2014) estimate price effects from an antitrust authority announcing an ongoing gasoline cartel investigation. The announcement reveals a commitment to regulatory action, while the focus of our study is on nonbinding signals that do not commit the antitrust authority to take future action.

2 Throughout, we discuss institutional details for understanding cost passthrough derived from a recent in‐depth industry report, ACCC (2020). For the 3 years our study focuses on, aside from the COVID‐19 world oil price shock, there are no other substantive changes to the industry.

3 “Oil Prices Dive as Saudi Arabia Takes Aim at Russian Production” (New York Times, March 8, 2020).

4 “Oil Plunge to Slash Petrol Prices, inflation” (The Australian Financial Review, March 9, 2020).

5 “We'll call them out”: Watchdog warning as Sydney Petrol Prices Vary by 65 Cents (Sydney Morning Herald, March 22, 2020).

6 In theory, if consumers are fully aware of prices and wholesale costs, there would be little scope for the static channel. This possibility is salient in our setting because of a state‐run gasoline price comparison platform that enables consumer search, which we discuss momentarily. However, using aggregated user statistics from the platform provided to us by the state government, following Byrne and de Roos (2021), we estimate that 22% of gasoline consumers use it when purchasing gasoline week‐to‐week. Thus, many consumers are not highly informed about high‐frequency station‐level gasoline prices through the platform. In addition, our estimated empirical models below reveal asymmetric passthrough of positive and negative wholesale shocks in the data, which is also in line with pricing behavior in the presence of search frictions (Tappata, 2009; Yang & Ye, 2008). This evidence underlines the static channel for announcement effects in our setting.

7 Barrage et al. (2020) illustrate how reputation can affect gasoline retailers' demand and profits. In particular, they show that BP's gasoline stations' margins and volumes declined following BP's major oil spill in 2010. The ACCC has a history of publishing in national media outlets rankings of gasoline retailers by average price levels. On October 17, 2019, the ACCC published retailer price rankings in the Sydney Morning Herald article ‘Petrol is petrol’: Coles Express is Country's Most Expensive. This history lends credibility to the ACCC's threats of “calling out” high‐priced retailers following the March 9 and 20 announcements, with potential future damage to their reputation, demand, and profits.

8 Without data on quantity sold at the market or station level, a frequent limitation in retail gasoline studies, we are unable to identify mechanisms for announcement effects. This is an area of future research.

9 We have trawled through Google News and Twitter over the March 21 period to look for other events that might drive these trends. There are no other news articles related to anticompetitive conduct in Australia except articles about retail gasoline and the government announcements on March 9 and 20. Moreover, there are no news cycles on world oil prices except those related to the March 9 and 20 announcements.

10 Sydney defined per its “Urban Centers and Localities” (UCLs) definition from the ABS. We use urban centers defined by UCLs for rural markets, which we validate using postal area definitions from Australia Post.

11 Newcastle (pop. 453,000) and Central Coast (pop. 337,000) are relatively more dense regions in NSW that also exhibit price cycles. Stations in the Gold Coast/Tweed Heads region in the state's north also exhibit price cycles but are part of the larger Gold Coast urban market in the neighboring state of Queensland. For the sake of brevity, we focus our urban market analysis on Sydney because it covers the vast majority of cycling stations in the state. Announcement effect estimates for Newcastle and Central Coast are available upon request.

12 Within urban markets in Australia, brands centrally set retail prices (ACCC, 2020). While there is some within‐brand variation in pricing across Sydney, it is dominated empirically by intertemporal price variation due to the price cycles where the timing and magnitude of station‐level price cuts and jumps are highly (but not perfectly) coordinated within a brand.

13 In addition, these retailers tend to initiate and lead price jumps while smaller independent retailers tend to follow. See, for example, Noel (2007), Lewis (2012), or Byrne and de Roos (2019) for extensive studies of price leadership and cycles in retail gasoline. While this is not the focus of the current study, we have undertaken analogous analyses of price leadership with our data. Results are available upon request.

14 Inventory adjustments or infrequent changes to station‐level operating costs (labor, convenience stores, and garages) can create lagged dependence between price and wholesale cost, particularly with rural stations. These factors underline the importance of using dynamic models that flexibly account for intertemporal linkages between price and wholesale costs to identify announcement effects.

15 The 49‐ and 21‐day lags for wholesale and retail prices are in line with previous studies on gasoline pricing dynamics (e.g., Borenstein et al., 1997; Lewis & Noel, 2011), allowing for substantial persistence in wholesale price shocks over time. All of our results are robust to variations in this lag structure.

16 We estimate $z_{it}$ by first regressing $p_{it}$ on $c_{it}$ and a set of station fixed effects, which corresponds to Equation (2). Importantly, the fixed effects account for, among other things, unobserved differences in station‐level transportation costs in wholesale prices that can generate dispersion in our price‐TGP margin measure across rural stations. With the estimated $\hat{\psi }$ and ${\hat{\nu }}_{i}$'s from (2), we compute $z_{it}$ and then estimate (1). Our bootstrap routine accounts for our two‐step estimation procedure in computing standard errors and confidence intervals.

17 An extensive out‐of‐sample forecasting analysis confirms our model's predictive ability. We estimate error‐correction models using subsamples of data before March 2020 and find that, regardless of subsample, our model accurately predicts future retail price changes out‐of‐sample. These results are available upon request.

18 There are three dates with restriction changes in our sample. On March 23, indoor venues such as pubs, gyms, cinemas, and casinos are closed in NSW. On March 30, the government imposed a state‐wide stay‐at‐home order. On May 15, the state‐wide stay‐at‐home order ended.

19 We measure traffic volume by the average number of passing vehicles recorded by counter stations within a 20‐km radius of a given station. The sample size changes reported in columns (2), (4), and (6) of Table 2 reflect the fact that 370 of our 668 rural stations have a traffic counter within a 20‐km radius. We likewise have different sample sizes for our Sydney‐based results below when our COVID controls are included in our models due to a subset of stations not having a traffic counter within a 20‐km radius.

20 For the sake of brevity, in what follows when we say *announcement effect* we mean an excessive price change relative to model predictions. Figure B.1 in the Supporting Information plots the realized and model‐predicted daily price changes that underly the statistically significant and negative coefficients on March 9 and 20 in Table 2. The figure shows realized and predicted daily price changes track closely on the days just before the announcements, in line with our identification strategy. However, realized and predicted price changes immediately diverge on March 9 and 20 as the realized (negative) price changes become larger in magnitude than the model's predicted price changes on these dates.

21 Heterogeneous announcement effects among high‐ and low‐priced stations may also arise from low‐priced stations having less scope to cut prices profitably. However, this channel is less relevant in our context because all high‐ and low‐priced stations realize large price‐cost margins. For instance, 94% and 63% of stations in rural markets and Sydney earn margins greater than 10 cpl on the announcement dates, which compares to a sample average wholesale cost of 122.9 cpl.

22 Other market characteristics to consider include the number of stations per capita, the share of independent stations, classifying markets depending on whether station i has other same‐branded retailers in the market, urban density, and demographics related to household income, age, or education. The inclusion of these variables in $X_{it}$ does not affect any of our main results, nor are they able to explain variation in announcement effects conditional on the main variables that we include in $X_{it}$.

23 We account for mean reversion in identifying announcement effects among high‐priced stations within local markets the day before the announcements. Recall that our model features error correction with station fixed effects, implying a station's price is constantly drifting toward its long‐run mean and its long‐run position within the market's price distribution each day. Relatively high‐priced stations just before announcements exhibiting substantive announcement effects reflect abnormal price adjustment on announcement dates above and beyond their predicted price change toward their ‘typical’ location within their market's price distribution given current and lagged wholesale prices and costs on announcement dates. Our Sydney‐based analysis below similarly accounts for mean reversion in identifying announcement effects among high‐priced stations.

24 We also examine time‐varying announcement effects among these retailers. We find Woolworths' stations exhibit excessive 1.31 cpl price cuts the day after the March 20 announcement. Woolworths's difference in passthrough could reflect different price elasticities among its consumers to whom they offer loyalty programs that tie grocery store sales to gasoline prices. In particular, Woolworths and Coles both offer 4‐cpl gasoline price discounts (3% of the 137 cpl average price in the sample) when consumers purchase more than $30 in groceries.

25 Working at the station‐level is important for accounting for station‐level differences in the timing and magnitude of price jumps and cuts. See, for example, Lewis (2012) or Byrne and de Roos (2019) which contains analyses of station‐level price dispersion over the cycle. Similar station‐level price dispersion of the cycle exist in our data.

26 We include indicators for margin thresholds as transitions to the relenting phase typically occur when margins are close to zero, thereby inducing nonlinear effects of margins on state transitions.

27 This distinguishes our approach from that of Lewis and Noel (2011) who observe prices at the market level and treat transitions between phases as unobserved. Price observations at the station level allow for direct observation of price jumps associated with the relenting phase. Figure B.2 in Supporting Information shows these cycling dynamics for an example station in Sydney.

28 We estimate only transitions from the undercutting phase to the relenting phase and assume that $\;Pr\;\left(s_{t}=0\;|\;s_{t-1}=1\right)=1$ as at the station level the vast majority of large price increases occur on a single day once per price cycle.

29 Accounting for local and temporal‐spatial dependence in errors in this way aligns with previous research on gasoline pricing (e.g., Chandra & Tappata, 2011), which reflects highly localized competition within urban retail gasoline markets (Chandra & Tappata, 2011; Houde, 2012). Indeed, the limited amount of within‐brand price dispersion across space reflects the importance of localized competition across Sydney.

30 An expanded table of coefficient estimates is provided in Table B.4.

31 As with the rural market analysis, we have also considered various other variables to include in $X_{it}$ for our Sydney‐based analysis including the number of stations within different radii of a given station, number and share of rival and commonly‐branded stations within different radii, and local HHI again within different radii. None of these local market structure variables affects our estimated announcement effects.

32 As in rural markets, the March 20 announcement effect is larger magnitude than the March 9 effect. As discussed above, the Treasurer's March 9 announcement may prime stations in advance of the March 20 announcement, or retailers may put different weight on the credibility of the Treasury versus the national antitrust authority in making threats.

33 As a further robustness check, we also estimate the model for all three phases with retailer interactions. These estimates are in Table B.6 in the Supporting Information.
