# Supplementary material for: Price effects of calling out market power: A study of the COVID‐19 oil price shock
Source: J Econ Manag Strategy. 2022 May 19:10.1111/jems.12485. Online ahead of print. doi: 10.1111/jems.12485 (PMC9347766; doi:10.1111/jems.12485)
Supplement: Supplementary file 1 — Supplementary Information [file JEMS-9999-0-s001.pdf]

Appendix

A COVID-19 Timeline in New South Wales

January 25 First confirmed case of COVID-19 in Australia.

February 28 Prime Minister Scott Morrison declares a pandemic, activating Australia's emergency health response plan.

March 2 First cases of local transmission in Australia confirmed in NSW.

March 9 Announcement 1: Treasurer Calls for Cost Pass-through

March 15 All overseas arrivals required to self-isolate for 14 days.

March 16 Non-essential gatherings of more than 500 people banned.

March 20 Announcement 2: ACCC Threatens Retailers

March 21 Travel ban on foreign nationals entering Australia. Queensland, Western Australia, Northern Territories, and South Australia close borders to non-essential travelers.

March 23 First major restrictions imposed: pubs, clubs, gyms, cinemas, casinos and religious gatherings shut down nationwide from midday. Restaurants and cafes restricted to takeaway and delivery.

March 24 Weddings limited to five people, funerals and outdoor gatherings to 10, and gatherings in people's homes to 10. Events and venues to be closed and banned expands to beauty and massage parlors, auctions and more.

March 28 All people entering Australia required to undertake a mandatory 14-day quarantine at designated facilities in their port of arrival.

March 30 Stay-at-home order in place. Both indoor and outdoor public gatherings limited to two persons only.

May 12 NSW Premier Gladys Berejiklian announced NSW will enter stage one of the relaxed restrictions from Friday May 15. These include an increase to 5 visitors per household, 10 customers in a café or restaurant, 10 guests at a wedding, 20 people at indoor funerals and 30 people at outdoor funerals.

June 1 Intrastate travel allowed, pubs and restaurants can have up to 50 customers, 20 people can attend weddings, museums, galleries, libraries and beauty salons will also reopen.

June 13 Households will be able to welcome up to 20 guests in addition to those already living in the house. Up to 20 people may gather in groups, food courts will be open as long as people can abide density requirements.

Sources: Coronavirus Disease (COVID-19) Weekly Epidemiology Report #11, Australia, 2020, www1.health.gov.au, Sydney Morning Herald 28-29 March 2020, SBS News App 12 May 2020, SBS News 1 June, ABC News 11 June

A.1 Traffic flows and gasoline demand

Table A.1: Summary Statistics of Daily Traffic Volume

	Sydney				Rural Markets			
	Mean	Std. Dev.	5th Pctl.	95th Pctl.	Mean	Std. Dev.	5th Pctl.	95th Pctl.
vol	21354.0	9958.7	7739.0	39137.0	21354.0	9958.7	7739.0	39137.0
Observations	187845				205633			
Counter Stations	225				236			

Notes: Traffic counts data are obtained from the NSW Roads Traffic Volume Counts API. The Traffic Volume Viewer map shows the locations of a selection of roadside counters across NSW. Table A.1 summarizes the daily number of vehicles passing a counter station in Sydney and the rural markets from January 1, 2017 to June 30, 2020.

Figure A.1: 7-Day Moving Average of the Counter-Level Daily Traffic Volume in Sydney

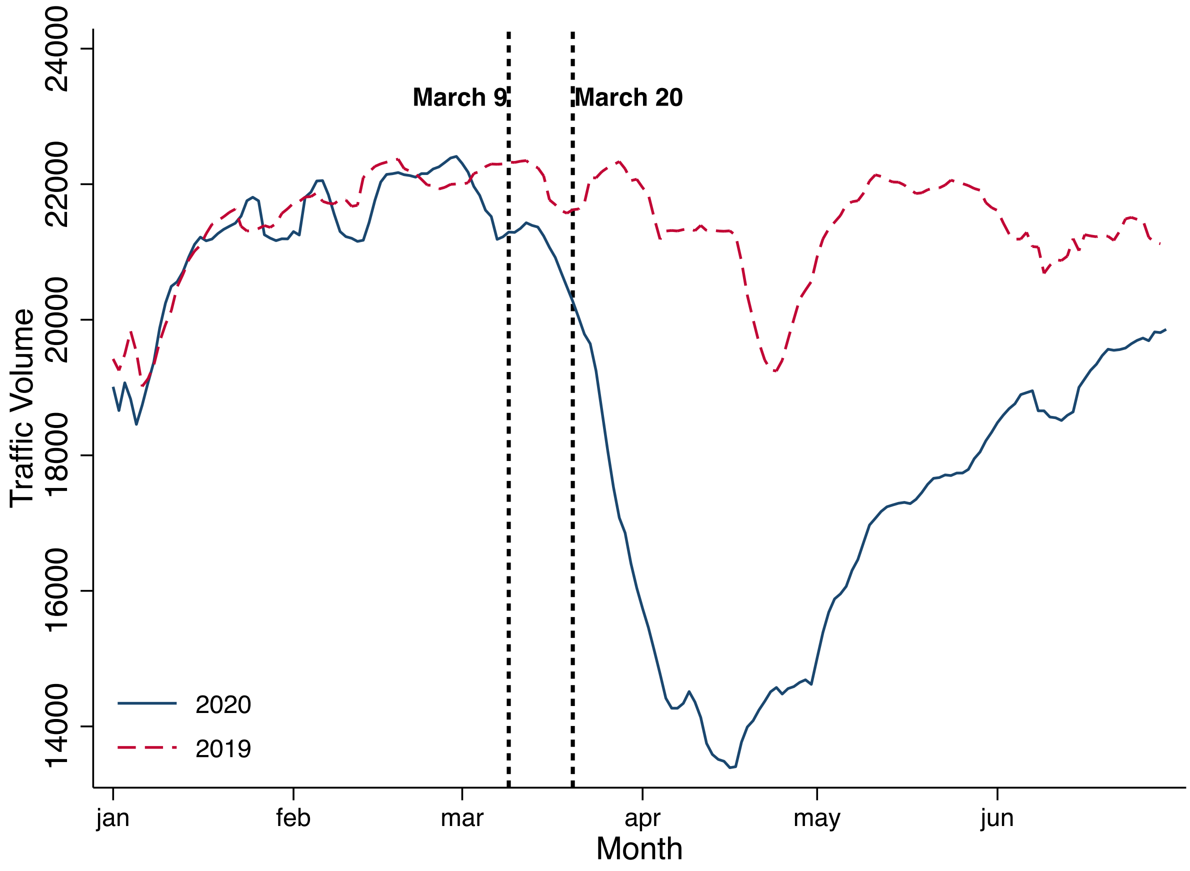

Notes: The daily average traffic volume is calculated over the 225 counter stations in Sydney. 7-day moving average plotted to eliminate day-of-week fluctuations in traffic volumes.

B Supplemental Figures and Tables

Figure B.1: Realized and Predicted Price Changes in Rural Markets Around Antitrust Announcements

(a) March 9 Announcement

(b) March 20 Announcement

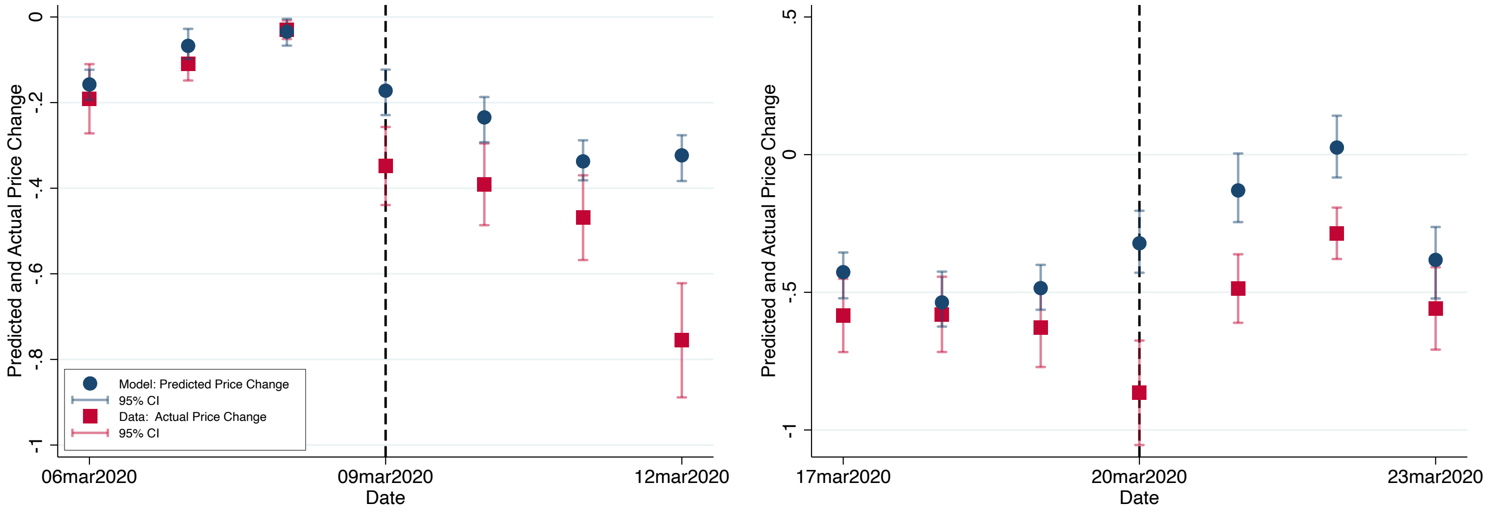

Notes: Figure B.1 breaks down the significant effects on the price following the March 9 and 20 announcements in Table 2 of the paper. The blue circles are the *realized* average daily price change among rural market stations. The red squares are the corresponding *predicted* average daily price change. Excessive pass-through exists whenever the red squares sit below the blue circles. Panels (a) and (b) respectively show the realized and predicted price changes are the same before the March 9 and 20 announcements. However, they diverge on the announcement dates, with the realized price changes becoming larger in magnitude (e.g., are more negative) than the predicted changes. These divergences between realized and predicted price changes on March 9 and 20 underpin the statistically significant negative announcement effects on price for March 9 and 20 in Table 2 of the paper.

Figure B.2: Example Sydney Station: 7-Eleven, Maroubra

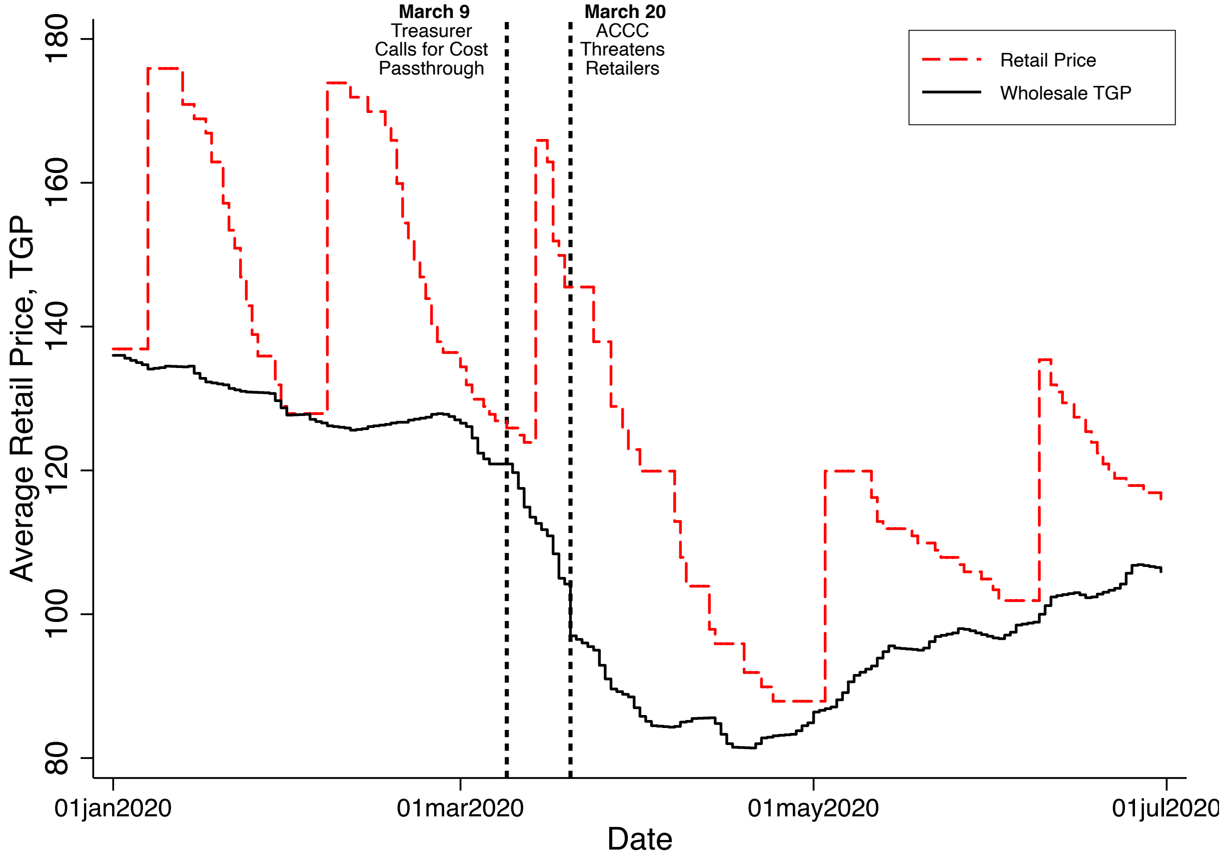

Table B.1: Parameter Estimates for the Error Correction Model for Rural Markets

	Positive		Negative	
	Coef.	SE	Coef.	SE
Δc_t	0.085***	(0.009)	0.007	(0.008)
Δc_{t-1}	0.030***	(0.008)	0.006	(0.007)
Δc_{t-2}	0.013	(0.008)	–0.015*	(0.008)
Δc_{t-3}	0.014*	(0.008)	–0.015**	(0.007)
Δc_{t-4}	0.028***	(0.008)	–0.051***	(0.007)
Δc_{t-5}	0.075***	(0.011)	0.002	(0.008)
Δc_{t-6}	0.061***	(0.008)	0.037***	(0.010)
Δc_{t-7}	0.081***	(0.010)	0.037***	(0.008)
Δc_{t-8}	0.031***	(0.010)	0.037***	(0.007)
Δc_{t-9}	–0.045***	(0.006)	–0.003	(0.008)
Δc_{t-10}	0.008	(0.007)	–0.018***	(0.006)
Δc_{t-11}	0.007	(0.009)	–0.008	(0.008)
Δc_{t-12}	–0.007	(0.007)	0.019***	(0.007)
...				
Δp_{t-1}	–0.059***	(0.013)	–0.064***	(0.011)
Δp_{t-2}	–0.025***	(0.005)	–0.031***	(0.010)
Δp_{t-3}	–0.036***	(0.006)	–0.020***	(0.004)
Δp_{t-4}	–0.024***	(0.006)	–0.008***	(0.003)
Δp_{t-5}	–0.029***	(0.007)	–0.009***	(0.003)
Δp_{t-6}	–0.020***	(0.004)	–0.001	(0.003)
Δp_{t-7}	0.004	(0.008)	0.024***	(0.008)
Δp_{t-8}	–0.007	(0.004)	–0.000	(0.004)
Δp_{t-9}	–0.006**	(0.003)	–0.014**	(0.007)
...				

Notes: Bootstrap standard error clustered at the market level is in parenthesis. The number of observation is 795022. $^{***}p < 0.01,^{**}p < 0.05,^{*}p < 0.1$

Table B.2: Summary Statistics for Estimates for the Station-specific Error Correction Term

	Mean	Std. Dev.	Min	Max
z_i^+	–0.021	0.019	–0.205	0.009
z_i^-	–0.022	0.019	–0.179	0.007
$STATION_i$	49.173	4.000	35.809	67.355

Notes: The number of stations is 668, where i is the station index.

Table B.3: Antitrust Announcements Effects Estimates in Rural Markets by Retailer

	(1)		(2)	
	March 9	March 20	March 9	March 20
BP	–0.19	–0.60**	–0.18	–0.23
	(0.16)	(0.29)	(0.18)	(0.30)
Caltex	–0.35***	–0.42*	–0.33**	0.03
	(0.11)	(0.22)	(0.13)	(0.24)
Coles	0.01	–0.88***	0.02	–0.49*
	(0.05)	(0.26)	(0.07)	(0.27)
Woolworths	0.05	0.22**	0.05	0.62***
	(0.04)	(0.09)	(0.05)	(0.16)
Metro	0.12	0.08	0.13	0.19
	(0.08)	(0.21)	(0.08)	(0.21)
United	–0.52**	0.20**	–0.51**	0.27**
	(0.21)	(0.10)	(0.21)	(0.13)
Independent	–0.16**	–0.73***	–0.15*	–0.45***
	(0.08)	(0.15)	(0.08)	(0.12)
Above Median Price			–0.04	–1.04***
			(0.12)	(0.27)
R-Squared	0.06		0.06	
Observations	795022		795022	

Notes: Regression coefficients on date fixed effects for March 9 and 20 in equation (1). The model is estimated on the full sample with and without the "Above Median Price" variable and interactions. Coefficients listed for each retailer and "Above Median Price" are interaction terms with the announcement dates. Standard errors clustered at the local market (UCL) level. $^{***}p < 0.01$, $^{**}p < 0.05$, $^{*}p < 0.1$.

Table B.4: Markov Switching Model Coefficient Estimates for Sydney

	Undercutting price		Relenting price		State transitions	
	Coeff.	Std. Err.	Coeff.	Std. Err.	Coeff.	Std. Err.
Margin_{it-1}	-0.040***	(0.002)	-0.734***	(0.019)	-0.187***	(0.004)
Wholesale cost (TGP)	0.002	(0.002)	0.044***	(0.004)	0.012***	(0.001)
Δc_1	0.039***	(0.009)	-0.011	(0.143)	0.163***	(0.020)
Δc_2	0.033***	(0.009)	-0.409*	(0.217)	0.013	(0.027)
Δc_3	-0.013	(0.010)	-0.456**	(0.202)	0.108***	(0.028)
Δc_4	-0.056***	(0.009)	-1.348***	(0.192)	-0.168***	(0.029)
Δc_5	-0.064***	(0.010)	-0.046	(0.202)	-0.365***	(0.036)
Δc_6	0.041***	(0.009)	-0.586***	(0.152)	-0.207***	(0.040)
Δc_7	0.055***	(0.012)	-0.521***	(0.143)	-0.118***	(0.042)
Δc_8	0.041***	(0.012)	-0.146	(0.169)	-0.075	(0.058)
Δc_9	0.040***	(0.013)	0.111	(0.135)	0.087*	(0.050)
Δc_{10}	0.005	(0.013)	0.332*	(0.169)	0.464***	(0.055)
Δc_{11}	0.025**	(0.011)	-0.315**	(0.148)	0.193***	(0.052)
Δc_{12}	0.022*	(0.012)	-0.654***	(0.161)	0.300***	(0.038)
Δc_{13}	0.053***	(0.014)	0.300	(0.203)	0.141***	(0.039)
Δc_{14}	0.054***	(0.013)	-0.959***	(0.217)	0.176***	(0.033)
Δc_{15}	0.066***	(0.010)	0.034	(0.232)	-0.105***	(0.026)
Δc_{16}	0.013	(0.014)	-0.160	(0.196)	-0.113***	(0.031)
Δc_{17}	-0.043***	(0.011)	-0.283	(0.265)	-0.010	(0.033)
Δc_{18}	-0.006	(0.012)	-0.718***	(0.217)	-0.052*	(0.027)
Δc_{19}	-0.023*	(0.013)	-1.054***	(0.261)	0.060**	(0.026)
Δc_{20}	-0.098***	(0.012)	-0.027	(0.205)	-0.128***	(0.031)
Δc_{21}	-0.085***	(0.013)	-0.782***	(0.202)	-0.166***	(0.027)
Δc_{22}	-0.039***	(0.012)	-0.077	(0.181)	-0.160***	(0.026)
Δc_{23}	-0.070***	(0.010)	-0.619***	(0.193)	-0.067**	(0.029)
Δc_{24}	-0.014	(0.011)	0.253	(0.156)	0.124***	(0.029)
Δc_{25}	0.019**	(0.009)	-0.360**	(0.162)	-0.030	(0.032)
Δc_{26}	-0.012	(0.011)	0.248	(0.184)	-0.057*	(0.033)
Δc_{27}	0.067***	(0.009)	0.192	(0.196)	0.101***	(0.029)
Δc_{28}	0.143***	(0.011)	-0.932***	(0.146)	0.261***	(0.024)
$\text{Margin}_{it-1} < 10$ cpl.					-0.121***	(0.045)
$\text{Margin}_{it-1} < 5$ cpl.					-0.230***	(0.032)
$\text{Margin}_{it-1} < 2$ cpl.					-0.099***	(0.033)
Observations	726325		31026		712632	

Notes: Coefficient estimates for the Markov switching model estimated on the full sample from Table 3, with standard errors in parentheses. $^{***}p < 0.01$, $^{**}p < 0.05$, $^{*}p < 0.1$

Table B.5: Markov Switching Model Coefficient Estimates for Sydney, Robustness Checks

	Undercutting price		Relenting price		State transitions	
	Full (1)	COVID (2)	Full (3)	COVID (4)	Full (5)	COVID (6)
Panel (a): main effects						
March 9	0.65***	0.36***	4.94***	5.53*	-0.89***	1.53***
	(0.07)	(0.08)	(1.57)	(2.87)	(0.27)	(0.31)
March 20	-0.31	-1.01***	0.28	-6.08	5.19***	7.71***
	(0.25)	(0.27)	(1.66)	(5.12)	(0.35)	(0.74)
R-Squared	0.25	0.24	0.93	0.96	0.18	0.33
Observations	726325	73723	31026	2296	712632	73115
Panel (b): effects by price level and local competitors						
March 9	0.75***	0.29***	2.66	3.47	-3.49***	-0.99
	(0.10)	(0.11)	(5.73)	(7.13)	(0.81)	(0.78)
March 20	0.52	0.00	1.30	-5.07	5.35***	8.22***
	(0.52)	(0.51)	(2.61)	(5.47)	(0.57)	(0.82)
Above Median Price	-0.38***	-0.37***	-0.42***	-0.25	-0.81***	-0.17
	(0.02)	(0.03)	(0.15)	(0.65)	(0.06)	(0.11)
March 9 × Above Median Price	0.05	0.14	4.01	3.77	2.14***	1.74***
	(0.10)	(0.10)	(4.47)	(5.14)	(0.48)	(0.49)
March 20 × Above Median Price	-2.52***	-2.75***				
	(0.32)	(0.33)				
Number of Stations within 1 mile	-0.15***	0.02	0.35	9.36***	0.04***	-0.81***
	(0.01)	(0.05)	(0.28)	(2.98)	(0.00)	(0.04)
March 9 × Number of Stations	-0.00	0.01	-0.12	-0.13	0.32***	0.31***
	(0.02)	(0.02)	(0.40)	(0.51)	(0.11)	(0.11)
March 20 × Number of Stations	0.08	0.09	-0.30	-0.22	-0.11	-0.11
	(0.09)	(0.08)	(0.38)	(0.43)	(0.11)	(0.10)
R-Squared	0.26	0.25	0.93	0.96	0.18	0.34
Observations	726325	73723	31026	2296	712369	72852

Notes: Coefficient estimates for the Markov switching model estimated on the full sample without a COVID-period fixed effect (odd columns), and estimated on only the COVID-period sample (even columns), with standard errors in parentheses. For the estimates on undercutting prices without a COVID-period fixed effect in column (1), announcement date coefficient estimates near zero indicate the presence of an announcement effect as the overall level of price declines is lower in this period compared to previous undercutting phases (i.e. most per-day coefficients δ_k are positive and significant, with an average value of 1.18). R-Squared denotes pseudo-R^2 for state transition models. $^{***}p < 0.01$, $^{**}p < 0.05$, $^{*}p < 0.1$

Table B.6: Antitrust Announcement Effects in Sydney by Retailer

	Undercutting price		Relenting price		State transitions	
	(1)	(2)	(3)	(4)	(5)	(6)
March 09 × Independent	-0.40**	-0.53***	3.42	-0.62	-7.11***	12.19***
	(0.19)	(0.20)	(3.14)	(5.16)	(1.24)	(0.95)
March 09 × 7-Eleven	0.03	-0.19	6.81**	2.68	-4.87***	14.42***
	(0.21)	(0.21)	(3.22)	(5.23)	(0.79)	(1.70)
March 09 × BP	0.07	-0.15	-5.07	-7.82	-5.84***	13.41***
	(0.22)	(0.24)	(10.28)	(10.01)	(0.97)	(1.12)
March 09 × Caltex	-0.19	-0.39*	5.69	1.57	-5.40***	13.62***
	(0.21)	(0.23)	(3.56)	(5.40)	(0.83)	(2.07)
March 09 × Woolworths	0.05	-0.16	8.45***	5.91	-5.15***	14.03***
	(0.21)	(0.23)	(3.18)	(4.10)	(0.83)	(0.95)
March 09 × Coles	-0.23	-0.50**	5.19	3.02	-4.38***	14.79***
	(0.20)	(0.22)	(3.78)	(4.34)	(0.78)	(1.98)
March 09 × Metro	-0.47**	-0.61**				
	(0.23)	(0.25)				
March 20 × Independent	0.14	-0.08	-6.79	-6.79	-0.66	19.36
	(0.36)	(0.39)	(4.46)	(4.42)	(0.64)	(.)
March 20 × 7-Eleven	-2.21***	-0.56	4.30	4.31	2.99***	23.11***
	(0.46)	(0.80)	(3.24)	(3.21)	(1.03)	(1.67)
March 20 × BP	-1.89**	-1.39*	1.61	1.60	1.17*	21.19
	(0.91)	(0.79)	(3.01)	(2.99)	(0.65)	(.)
March 20 × Caltex	-1.98***	-0.52	-6.67	-6.66	0.71	20.74***
	(0.52)	(0.71)	(6.71)	(6.66)	(1.20)	(1.85)
March 20 × Woolworths	-1.48***	0.01	1.18	1.15	3.42***	23.59***
	(0.38)	(0.67)	(3.69)	(3.68)	(1.22)	(1.57)
March 20 × Coles	-1.32***	-0.09	2.22	2.03	4.44***	24.77***
	(0.38)	(0.61)	(3.07)	(3.04)	(0.73)	(2.01)
March 20 × Metro	0.69*	0.36				20.02***
	(0.39)	(0.40)				(2.11)
March 09 × Above Median Price		0.04		4.46		1.39***
		(0.09)		(4.13)		(0.48)
March 20 × Above Median Price		-2.31***				
		(0.62)				
Observations	726325	726325	31026	31026	712558	712912

Notes: Dependent variable is undercutting phase price changes. The model is estimated on the full sample with and without the "Above Median Price" variable and interactions. Standard errors clustered at the post-code level. *** $p < 0.01$, ** $p < 0.05$, * $p < 0.1$
